# A retrospective paired study: efficacy and toxicity of nimotuzumab versus cisplatin concurrent with radiotherapy in nasopharyngeal carcinoma

**DOI:** 10.1186/s12885-016-2974-x

**Published:** 2016-12-13

**Authors:** H. M. Li, P. Li, Y. J. Qian, X. Wu, L. Xie, F. Wang, H. Zhang, L. Liu

**Affiliations:** 1 State Key Laboratory of Biotherapy and cancer center, West China Hospital, Sichuan University, Chengdu, 610041 China; 2 Department of Cardiovascular Surgery, West China Hospital, Sichuan University, Chengdu, 610041 China; 3 Department of Medical Oncology Cancer Center, State Key Laboratory of Biotherapy, West China Hospital, Sichuan University, Chengdu, 610041 China

**Keywords:** Nasopharyngeal carcinoma, Nimotuzumab, Radiotherapy, Efficacy, Toxicity

## Abstract

**Background:**

To compare efficacy and toxicity of nimotuzumab versus cisplatin (CDDP) concurrent with intensity modulated radiation therapy (IMRT) in patients with nasopharyngeal carcinoma (NPC).

**Methods:**

We retrospectively reviewed patients with NPC from September 2008 to November 2013. The synchronous regimens included h-R3/RT (nimotuzumab and radiotherapy) one time per week for 6–8 weeks and CDDP/RT (cisplatin and radiotherapy) every three weeks for 2–3 cycles. All patients in our analysis completed the planned IMRT and received TPF (docetaxel + cisplatin + 5-fluorouracil) neoadjuvant chemotherapy for two cycles.

**Results:**

Among the 302 NPC patients who were treated definitively with TPF neoadjuvant chemotherapy followed by IMRT concurrent with nimotuzumab or cisplatin at West China Hospital Sichuan University, 52 patients received h-R3/RT with complete clinical and follow-up data. Based on age, sex and tumor stage, 104 eligible patients were propensity-matched, with 52 patients in each treatment group (h-R3/RT and CDDP/RT). With a median follow-up of 50 months, the 5-year overall survival (OS) and progression-free survival (PFS) rates for the h-R3/RT vs. CDDP/RT treatment groups were 63.9% vs. 81.4% (*p* = 0.024) and 58.0% vs. 80.6% (*p* = 0.028), respectively. The h-R3/RT patients experienced less leukopenia and milder nausea and vomiting. In our sub-analysis, for stage II patients, no significant differences were found in OS and PFS, whereas milder nausea and vomiting were found in the h-R3/RT group (*p* = 0.046). Moreover, for patients older than 60 years, there were no statistically significant differences in OS and PFS, whereas milder nausea and vomiting was observed in the h-R3/RT group (*p* = 0.020).

**Conclusions:**

Although CDDP/RT remains the preferred choice for most patients with NPC, h-R3/RT may be a treatment option for the patients with stage II, older than sixty years old, and who are intolerable to cisplatin.

**Electronic supplementary material:**

The online version of this article (doi:10.1186/s12885-016-2974-x) contains supplementary material, which is available to authorized users.

## Background

Nasopharyngeal carcinoma (NPC) is endemic in Southern China with an annual incidence of 25 cases per 100,000 [1]. A total of 95% of NPC cases are non-keratinizing (differentiated and undifferentiated included). It has been widely known that NPC is closely associated with EB viral infection, and its pathogeny includes environmental factors and genetic susceptibility [2]. Radiotherapy comprises the foundational treatment for nasopharyngeal cancer. In 1998, Al-Sarraf M et al. reported the results of INT 0099 (a phase III clinical trial), making concurrent chemoradiotherapy (CCRT) as the standard treatment for locally advanced nasopharyngeal cancer [3]. The National Comprehensive Cancer Network (NCCN) has recommended concurrent chemoradiotherapy with or without adjuvant chemotherapy for NPC patients with stage II–IV according to several prospective randomized trials [4–7].

Radiotherapy induced mucositis and weight loss, which cause the physical deterioration of patients. Oral mucositis is sustained for 2–3 weeks after radiotherapy, which results in the reduction of tolerance and compliance with adjuvant chemotherapy and which is associated with poor efficacy of treatment [8]. These facts have gradually made the regimen of concurrent chemoradiotherapy plus adjuvant chemotherapy undesirable. Theoretically, conventional concurrent chemotherapy can improve the local control rate, but its role for eliminating subclinical metastases is very limited in clinical practice. Clinically, neoadjuvant chemotherapy can improve the efficacy and reduce the rate of distant metastasis [9], thus it has been universally applied of neoadjuvant chemotherapy plus concurrent chemoradiotherapy for locally advanced nasopharyngeal carcinoma. Increasing amounts of data from randomized clinical trials have recommended cisplatin as the drug for use in concurrent chemoradiotherapy [10, 11]. However, despite the increasing of beneficial antitumor effects, cisplatin concurrent with radiotherapy also elevates the occurrence of severe toxicities, which include marrow suppression, nausea and vomiting, which commonly are intolerable to most patients with nasopharyngeal carcinoma [12]. Therefore, it is urgent to explore more effective and tolerable regimens for NPC.

Over-expression of epidermal growth factor receptor (EGFR) gene amplifications is associated with many types of cancers, including NPC [13], and the positive-expression rate of EGFR is more than 90% in non-keratinizing NPC [14, 15]. Altered EGFR signaling is widely implicated in cell apoptosis resistance, proliferation, radiotherapy resistance, metastasis and invasion [16, 17]. Targeted therapies for treatment of NPC have become a topic of increased research interest internationally due to favorable efficacy and low toxicity. Nimotuzumab is a humanized anti-EGFR mouse monoclonal antibody designed to reduce immunoreactivity and to enhance radio sensitivity [18]. Earlier clinical trials of nimotuzumab concurrent with radiotherapy in patients with locally advanced head and neck squamous cell carcinoma reported that this combination therapy was well tolerated and may enhance the radio curability of unresectable head and neck neoplasms [19]. A multi-center, randomized controlled phase II clinical study was performed to observe the efficacy and adverse reactions of nimotuzumab combined with radiotherapy for advanced nasopharyngeal carcinoma, led by the Chinese Academy of Medical Sciences. The results showed that the 3-year overall survival of the group treated with nimotuzumab combined with radiotherapy was 84.29%, significantly higher than the group treated with radiotherapy alone (77.61%) [20], which suggested a synergistic effect between nimotuzumab and radiotherapy. Its side effects were mild, and it did not affect the normal execution of radiotherapy. Moreover, nimotuzumab combined with radiotherapy was recommended in the 2010 version of the Chinese head and neck cancer practice guidelines. However, the efficacy and toxicity of nimotuzumab concurrent with radiotherapy compared with cisplatin concurrent with radiotherapy for the treatment of patients with NPC remains an area of uncertainty.

In this study, we aimed to shed light on this issue. The primary endpoint was the evaluation of overall survival and progression-free survival. Secondary endpoints included the assessment of toxicity, including hematological toxicity, liver function, dermatitis, rash, mucositis, taste change, vomiting, and weight loss.

## Methods

From September 2008 to November 2013, 302 patients with NPC treated definitively with nimotuzumab or cisplatin concurrent with IMRT at West China Hospital, Sichuan University were included. The main study endpoint was efficacy (OS, PFS), and the secondary endpoints were toxicities.

### Patients

A retrospective review was conducted using the case records of patients with NPC treated at West China Hospital Sichuan University from September 2008 to November 2013. Our research retrospectively analyzed the clinical routine data, and was granted an exemption from requiring ethics approval by the Subcommittee on Biomedical Ethics of West China Hospital, Sichuan University. Patients were eligible for this study if they met the following inclusion criteria: patients with NPC were pathologically confirmed at West China Hospital, the patients did not receive any antitumor therapy before admission, the intensity modulated radiation therapy (IMRT) was administered at West China Hospital, all patients received TPF neoadjuvant chemotherapy two cycles before radiotherapy, synchronous regimens included h-R3/RT (nimotuzumab and radiotherapy) and CDDP/RT (cisplatin and radiotherapy), and the patient’s ECOG score was less than or equal to 2 points. The another reason why patients selected to receive nimotuzumab rather than cisplatin was that they could not tolerate the side effects (nausea and vomiting) caused by neoadjuvant chemotherapy. Before treatment, doctors introduced the evidence-based medicinal benefits and side effects of nimotuzumab, and all patients signed informed consent. Among the 302 patients, there were 52 cases with complete clinical and follow-up data and 7 cases without complete clinical and follow-up data in the h-R3/RT group, and there were 221 cases with complete clinical and follow-up data and 22 cases without complete clinical and follow-up data in CDDP/RT group. Due to the significant differences in general information, we included 52 pairs based on age, sex and tumor stage (2010 7th edition AJCC staging classification) [21]. We selected the individual CDDP/RT patients paired with 52 h-R3/RT patients with complete clinical and follow-up data one by one, according to the following conditions: first, the age difference within the pair was less than 5 years; second, tumor stage, depth of invasion (T), lymph node metastasis (N), distant metastasis (M), clinical stages were consistent in the h-R3/RT patients as much as possible; third, the difference between the number of men and women was not more than 10; and finally, nonparametric tests were used to ensure that there was no difference in paired factors.

### Treatment

#### Neoadjuvant chemotherapy

Eligible patients received two cycles TPF neoadjuvant chemotherapy (docetaxel 75 mg/m^2^ d1 + DDP 25 mg/m^2^ d1-3 + 5-fu 600 mg/m^2^ d1-5). The schedule, dosage and duration were similar to Kong Lin’s study [22]. Radiotherapy started three weeks after the two neoadjuvant cycles.

#### Radiation therapy

The radioactive source was a medical linear accelerator (6 MV X). All patients received intensity modulated radiotherapy (IMRT) with a 2.12 or 2.24 Gy (skull base bone destruction) fraction once a day for 5 days per week up to a total of 70 or 74 Gy (skull base bone destruction) in 33 fractions. The delineation of target volumes was based on imaging (CT, MRI or FDG- PET), and the target volumes were performed in the same series. The technique and dose of radiotherapy was consistent with the principles of the NCCN guidelines.

#### Synchronous regimens

Nimotuzumab (200 mg) was administered once weekly for 6–8 weeks, and cisplatin (25 mg/m^2^) was administered every three weeks (d1-3) for 2–3 cycles according to the NCCN guidelines. The 200 mg dose of nimotuzumab was selected because this dose was reported to be as effective as 400 mg [19, 23]. Nimotuzumab or cisplatin were administered concurrent with IMRT.

#### Antiemetic

We selectively used antiemetic therapy to reduce gastrointestinal reactions depending on the severity of nausea and vomiting in patients of the two groups. Because the details of antiemetic use had been lost, it was impossible to calculate the relevant statistics. The antiemetic administered in our study included 5-HT3 antagonist (ondansetron/granisetron/tropisetron), a dopamine receptor antagonist (metoclopramide), an H1 receptor antagonist (diphenhydramine), and a proton pump inhibitor (pantoprazole).

### Follow-up

Via telephone or outpatient clinic visit, we recorded survival, recurrence and metastasis, and side effects, including hematological toxicities, liver function, dermatitis, rash, mucositis, altered taste, nausea and vomiting, weight loss and so on. Hematologic toxicity mainly comprised bone marrow suppression, including leukopenia, anemia and thrombocytopenia. Abnormal liver function was mainly manifested by elevated liver enzymes. Blood routine, biochemical routine and other toxicities were estimated at least once a week. Side effects were evaluated according to the CTCAE (Common Terminology Criteria for Adverse Events) 4.0 criteria.

After completion of treatments, the patients were subsequently reexamined every three months for two years, then every six months for the next three years, and annually thereafter to assess tumor status. The reexaminations included assessments of blood toxicity, EB virus DNA, pharyngorhinoscopy and biopsy, nasopharynx and neck MRI, chest CT, abdominal ultrasound and bone EC, etc. The follow-up time was calculated from the date of diagnosis of nasopharyngeal carcinoma to the date of death or last follow-up time.

### Statistical analysis

All statistical analyses were performed using IBM SPSS 20.0 software, and the tests were considered significant at *p* ≤ 0.05. The Kaplan–Meier method was used to estimate the 95% confidence intervals (95% CIs) of overall survival and progression-free survival. Overall survival was calculated from the date of diagnosis to the date of death from any cause or the last scheduled visit. Progression-free survival was defined as the time from diagnosis to the time of tumor progression or the last scheduled visit. Survival distributions were compared using a log-rank test. Toxicities were estimated by a paired rank sum test. Univariate analysis was performed using COX regression. Multivariate analysis using the COX proportional hazards model was used to calculate the hazard ratio (HR) with 95% CIs and to adjust for independent potential prognostic factors. The following potentially prognostic factors were considered in the multivariate analysis according to the results of univariate analysis and those mentioned in previous studies: age, sex, T stage, N stage, AJCC stage, anemia, dermatitis, nausea and vomiting, weight loss and drug. Assignment expressions of the factors in this research are listed in Additional file 1: Table S4.

## Results

### Patient characteristics

The patients and tumor characteristics of 104 cases are summarized in Table 1,and the general information of total 302 cases was listed in Additional file 1: Table S7. One hundred and four eligible patients were propensity-matched, with 52 patients in each group, and the median follow-up time was 50 months (range 12–74 months). Pathological type of the patients was non-keratinizing. All patients received the entire treatment IMRT course with the prescribed dose and two cycles of TPF neoadjuvant chemotherapy, and none of them underwent adjuvant chemotherapy after radiotherapy. A total of 46 (88.5%) and 6 patients (11.5%) in h-R3/RT group received 8 and 6 doses of nimotuzumab, respectively. And a total of 44 (84.6%) and 8 patients (15.4%) in CDDP/RT group received 3 and 2 cycles of cisplatin, respectively. Patient compliance is reported in Additional file 1: Table S5. According to the 2010 AJCC staging classification (7th edition) for nasopharyngeal cancer [21], patients in our study were divided into stage II (26 patients; 25.0%), III (41 patients; 39.4%) and non-metastatic stage IV (37 patients; 35.6%). There were no significant differences among the following variables: age (<60 vs. ≥ 60), sex, T stage, N stage, AJCC stage and ECOG scores (all *p* values > 0.05).

**Table 1:** Patients and tumor characteristics of the h-R3/RT and CDDP/RT groups (*N* = 104)

	h-R3/RT (*N* = 52)	CDDP/RT (*N* = 52)	
Characteristics	[n (%)]	[n (%)]	*P* Value*
Age			1.000
< 60	45 (86.5%)	45 (86.5%)	
≧60	7 (13.5%)	7 (13.5%)	
Sex			0.083
Male	42 (82.7%)	36 (69.2%)	
Female	10 (17.3%)	16 (30.8%)	
T category			0.799
1	11 (21.2%)	11 (21.2%)	
2	13 (25.0%)	14 (26.9%)	
3	12 (23.1%)	12 (23.1%)	
4	16 (30.7%)	15 (28.8%)	
N category			0.957
0	3 (5.8%)	4 (7.7%)	
1	20 (38.4%)	19 (36.5%)	
2	23 (44.3%)	23 (44.3%)	
3	6 (11.5%)	6 (11.5%)	
M category			1.000
0	52 (100%)	52 (100%)	
1	0 (0.00%)	0 (0.00%)	
Clinical stage			0.831
II	13 (25.0%)	13 (25.0%)	
III	20 (38.5 0%)	21 (40.4%)	
IV (non-metastatic)	19 (36.5.0%)	18 (34.6%)	
Histologic type			
non-keratinizing	52 (100%)	52 (100%)	
Total RT	(70/74)^a^	(70/74)^a^	
dose (Gy)			
ECOG	0-2	0-2	

*Abbreviations*: *RT* radiotherapy, *h-R3/RT* nimotuzumab and radiotherapy, *CDDP/RT* cisplatin and radiotherapy. *ECOG* Eastern Cooperative Oncology Group

^a^a total of 74 Gy when patients had skull base bone destruction

*All *p* values were obtained by the non-parametric test

### Efficacy

**Fig. 1:**
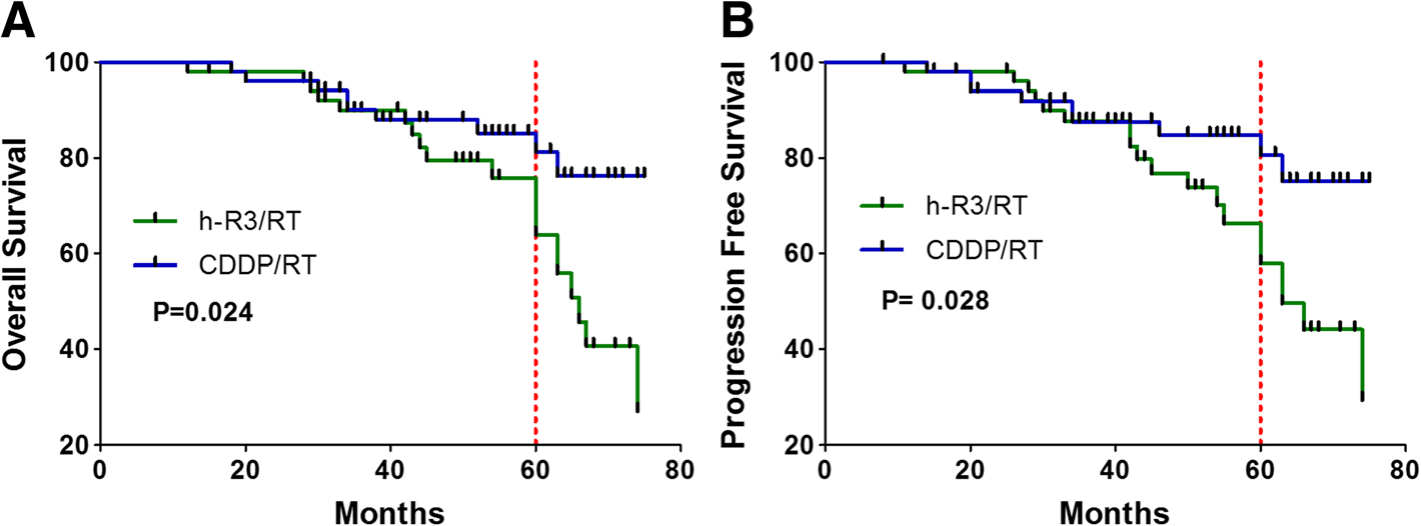
Overall survival (**a**) and progression-free survival (**b**) of patients who received h-R3/RT (*N* = 52) and CDDP/RT (*N* = 52) treatment

At a median follow-up of 50 months (range 12–74 months) for living patients, the 5-year OS and PFS rates of h-R3/RT and CDDP/RT group were 63.9% vs. 81.4% (*p* = 0.024) and 58.0% vs. 80.6% (*p* = 0.028), respectively. The survival curves were shown in Fig. 1.

Among the NPC patients with stage II AJCC, the OS of the two groups were not significantly different (*p* = 0.571). Furthermore, for the patients aged 60 years or older, there were no significant differences in OS (*p* = 0.236). The survival curves are shown in Additional file 2: Figures S1 and S2.

### Toxicity

The treatment toxicities resulting from the synchronous drugs were generally mild, and no fatal toxicity reaction occurred among all patients. Leukopenia was the most common hematological toxicity, and it was not accompanied by any serious infections. It was rare to find a specific toxicity that was induced by nimotuzumab, while it was common to suffer from nausea and vomiting in patients who received cisplatin.

Using paired rank sum test, we found significant differences in toxicities between two groups: leukopenia, nausea and vomiting. Patients in the h-R3/RT group experienced less leukopenia, and milder nausea and vomiting with p values of 0.048 and 0.000, respectively (Table 2). Oral mucositis was one of the most common RT-related toxicities, grade 3 or 4 oral mucositis as observed in 25 (48.1%) and 30 (57.7%) patients in the CDDP/RT and h-R3/RT groups, respectively. While grade 3 or 4 dermatitis was observed in 1 (1.9%) and 2 (3.8%) patients in the CDDP/RT and h-R3/RT groups, respectively. In total, 47 (90.4%) and 46 (88.5%) patients experienced taste change the CDDP/RT and h-R3/RT groups, respectively. Most patients in our study experienced weight loss, and grade 3 or 4 weight loss was observed in 15 (28.8%) and 13(25.0%) patients in the CDDP/RT and h-R3/RT groups, respectively. In our study, no patients required RT interruptions or terminations because of acute toxicity. Then, we analyzed toxicities among patients in stage II AJCC and found milder nausea and vomiting in the h-R3/RT group (*p* = 0.046) (Table 3). Moreover, as depicted in Table 4, among patients older than 60 years, a significant difference was also found in some toxicities, as patients in the h-R3/RT group experienced milder nausea and vomiting (*p* = 0.020).

**Table 2:** Toxicities of the h-R3/RT and CDDP/RT groups (*N* = 104)

Toxicities (RTOG Grade)	h-R3/RT (*N* = 52) [n (%)]	CDDP/RT (*N* = 52) [n (%)]	*P* Value*
WBC			0.048*
(1&2)	32 (61.5%)	32 (61.5%)	
(3&4)	1 (1.9%)	7 (13.5%)	
PLT			0.752
(1&2)	19 (36.5%)	18 (34.6%)	
(3&4)	1 (1.9%)	2 (3.8%)	
HB			0.499
(1&2)	27 (51.9%)	26 (50%)	
(3&4)	0 (0.00%)	0 (0.00%)	
ALT			0.294
(1&2)	10 (19.1%)	8 (11.9%)	
(3&4)	0 (0.00%)	0 (0.00%)	
AST			0.197
(1&2)	10 (19.2%)	5 (9.6%)	
(3&4)	0 (0.00%)	0 (0.00%)	
GGT			0.166
(1&2)	9 (17.3%)	5 (9.6%)	
(3&4)	0 (0.00%)	0 (0.00%)	
Dermatitis			0.445
(1&2)	33 (63.5%)	44 (84.6%)	
(3&4)	2 (3.8%)	1 (1.9%)	
Rash			0.248
(1&2)	5 (9.6%)	3 (5.8%)	
(3&4)	1 (1.9%)	0 (0.00%)	
Mucositis			0.093
(1&2)	21 (40.4%)	26 (50%)	
(3&4)	30 (57.7%)	25 (48.1%)	
Taste change			0.763
yes	46 (88.5%)	47 (90.4%)	
no	6 (11.5%)	5 (9.6%)	
Vomit			0.000*
only nausea	12 (23.1%)	2 (3.8%)	
nausea and vomit	5 (9.6%)	43 (78.8%)	
Weight loss			0.249
(1&2)	26 (50.0%)	30 (57.7%)	
(3&4)	13 (25.0%)	15 (28.8%)	

*Abbreviations*: *RT* radiotherapy, *h-R3/RT* nimotuzumab and radiotherapy, *CDDP/RT* cisplatin and radiotherapy. *WBC* white blood cell, *PLT* Platelets, *HB* hemoglobin, *ALT* alanine transaminase, *AST* glutamic-oxalacetic transaminease, *GGT* gamma glutamyl transpeptidase

*All *p* values were obtained by the paired rank sum test

**Table 3:** Toxicities in stage II patients who received h-R3/RT or CDDP/RT (*N* = 26)

Toxicities (RTOG Grade)	h-R3/RT (*N* = 13) [n (%)]	CDDP/RT (*N* = 13) [n (%)]	*P* Value*
WBC			0.711
(1&2)	10 (76.9%)	6 (46.2%)	
(3&4)	0 (0.0%)	3 (23.1%)	
PLT			0.380
(1&2)	6 (46.2%)	6 (46.2%)	
(3&4)	0 (0.00%)	1 (7.7%)	
HB			0.083
(1&2)	8 (61.5%)	5 (61.5%)	
(3&4)	0 (0.0%)	0 (0.0%)	
ALT			0.655
(1&2)	2 (15.4%)	3 (23.1%)	
(3&4)	0 (0.0%)	0 (0.0%)	
AST			0.157
(1&2)	2 (15.4%)	0 (0.0%)	
(3&4)	0 (0.0%)	0 (0.0%)	
GGT			1.000
(1&2)	1 (7.7%)	1 (7.7%)	
(3&4)	0 (0.0%)	0 (0.0%)	
Dermatitis			0.414
(1&2)	8 (61.5%)	11 (84.6%)	
(3&4)	0 (0.0%)	0 (0.0%)	
Rash			0.655
(1&2)	3 (23.1%)	2 (15.4%)	
(3&4)	0 (0.0%)	0 (0.0%)	
Mucositis			0.093
(1&2)	5 (38.5%)	8 (61.5%)	
(3&4)	7 (53.8%)	5 (38.5%)	
Taste change			1.000
yes	13 (100%)	13 (100%)	
no	0 (0.0%)	0 (0.0%)	
Vomit			0.046*
only nausea	6 (46.2%)	3 (23.1%)	
Nausea and vomit	2 (15.4%)	10 (76.9%)	
Weight loss			0.305
(1&2)	8 (61.6%)	7 (53.8%)	
(3&4)	3 (23.1%)	5 (38.5%)	

*Abbreviations: RT* radiotherapy, *h-R3/RT* nimotuzumab and radiotherapy, *CDDP/RT* cisplatin and radiotherapy

*All *p* values were obtained using the paired rank sum test

**Table 4:** Toxicities in patients aged more than 60 years who received h-R3/RT or CDDP/RT. (*N* = 14)

Toxicities (RTOG Grade)	h-R3/RT (*N* = 7) [n (%)]	CDDP/RT (*N* = 7) [n (%)]	*P* Value*
WBC			0.102
(1&2)	3 (42.9%)	5 (71.4%)	
(3&4)	0 (0.00%)	0 (0.00%)	
PLT			0.059
(1&2)	5 (71.4%)	1 (14.3%)	
(3&4)	0 (0.00%)	0 (0.00%)	
HB			0.157
(1&2)	2 (28.6%)	5 (71.4%)	
(3&4)	0 (0.00%)	0 (0.00%)	
ALT			0.317
(1&2)	0 (0.00%)	1 (14.3%)	
(3&4)	0 (0.00%)	0 (0.00%)	
AST			0.317
(1&2)	0 (0.00%)	1 (14.3%)	
(3&4)	0 (0.00%)	0 (0.00%)	
GGT			1.000
(1&2)	0 (0.00%)	0 (0.00%)	
(3&4)	0 (0.00%)	0 (0.00%)	
Dermatitis			0.317
(1&2)	4 (57.1%)	7 (100%)	
(3&4)	0 (0.00%)	0 (0.00%)	
Rash			1.000
(1&2)	0 (0.00%)	0 (0.00%)	
(3&4)	0 (0.00%)	0 (0.00%)	
Mucositis			0.891
(1&2)	2 (28.6%)	3 (42.9%)	
(3&4)	4 (57.1%)	2 (52.4%)	
Taste change			1.000
yes	5 (71.4%)	6 (85.7%)	
no	2 (28.6%)	1 (14.3%)	
Vomit			0.020
only nausea	0 (0.00%)	1 (2.4%)	
Nausea and vomit	0 (0.00%)	5 (71.4%)	
Weight loss			0.174
(1&2)	2 (28.6%)	4 (57.1%)	
(3&4)	1 (14.3%)	2 (26.2%)	

*Abbreviations*: *RT* radiotherapy, *h-R3/RT* nimotuzumab and radiotherapy, *CDDP/RT* cisplatin and radiotherapy

* All *p* values were obtained by use of the paired rank sum test

### Patterns of relapse and metastasis

The patterns of treatment failure and causes of death are summarized in Table 5. At the median follow-up of 50 months, there were 19 deaths. At the time of the analysis, two patient had locoregional failure, two patient showed locoregional failure and distant metastases, and ten patients developed distant metastases.

**Table 5:** Patterns of relapse of all patients who received h-R3/RT or CDDP/RT. (*N* = 104)

	h-R3/RT (*N* = 52)	CDDP/RT (*N* = 52)
Relapse and metastasis	[n (%)]	[n (%)]
Local recurrence	2 (3.9%)	2 (3.9%)
Hepatic metastases	2 (3.9%)	3 (5.8%)
Lung metastasis	1 (1.9%)	3 (5.8%)
Bone metastasis	1 (1.9%)	2 (3.9%)

*Abbreviations*: *RT* radiotherapy, *h-R3/RT* nimotuzumab and radiotherapy, *CDDP/RT* cisplatin and radiotherapy

### Prognosis

The overall survival (OS) of 104 cases were analyzed by univariate and multivariable COX, which were listed in Additional file 1: Table S1 and Additional file 1: Table S2, respectively. Based on results of previously reported studies and on the results of the univariate analysis, we included sex, age, T category, N category, clinical stage and side effects in the COX analysis. The results of univariate COX analysis showed that T category, N category, clinical stage, vomiting and drug were prognostic factors for nasopharyngeal carcinoma (Additional file 1: Table S1). Furthermore, age, N category and vomiting were indicated as independent prognostic factors for nasopharyngeal carcinoma according to the multivariable COX analysis. (Additional file 1: Table S2).

## Discussion

This study is a retrospective analysis of our institution’s history of treating nasopharyngeal carcinoma with h-R3/RT compared to radiotherapy and platinum-based chemotherapy (CDDP/RT). We followed up 104 patients, and at the median of 50 months, the 5-year OS and PFS rates were 63.9% vs. 81.4% (*p* = 0.024) and 58.0% vs. 80.6% (*p* = 0.028), respectively. CDDP/RT achieved better survival, while the patients who received h-R3/RT experienced less toxicity. It was suggested that, in patients who were greater than 60 years of age with stage II, there was no significant difference in survival, while h-R3/RT patients exhibited lower side effects.

To the best of our knowledge, this study is the first study that compared the efficacy and toxicities of nimotuzumab versus cisplatin concurrent with IMRT in nasopharyngeal carcinoma patients. In our study, the 5-year OS and PFS rates were 63.9% vs. 81.4% (*p* = 0.024) and 58.0% vs. 80.6% (*p* = 0.028), respectively. Al-Sarraf M et al. reported that the 3-year survival rate for patients who received CDDP/RT was 78% [3]. The difference in the survival rate may be caused by different radiotherapy techniques. Our study used IMRT, which is a more advanced radiotherapy technique. In a phase II clinical study, the long-term follow-up results failed to show a significant difference between h-R3/RT and radiotherapy alone in the long-term metastasis rate and survival rate [24]. The 3-year OS rates of the two groups dropped to 94.4 and 88.2%, respectively (*p* = 0.518). Thus, the strength of nimotuzumab combined with radiotherapy in NPC may be still largely due to a strengthening of the radiation antitumor effect.

Our study also found significant differences in toxicities between the h-R3/RT and CDDP/RT group. More leukopenia and heavier nausea and vomiting emerged in the CDDP/RT group, which might result from cisplatin-induced inherent hematologic toxicities and heavier gastrointestinal reactions [25].

Anti-EGFR-targeted therapy has become an important aspect of cancer treatment in recent years. Compared with other EGFR inhibitors, nimotuzumab shows a greater advantage in terms of less toxicity. For example, the toxicities of cetuximab include acne-like skin rash, itching, fever, nausea and so on [26]. With its humanized degree over 90%, nimotuzumab remarkably reduces human anti-mouse antibody and allergic reactions. Consistently, in our study, there were only three patients who suffered from acne-like skin rash in the h-R3/RT group. In the CDDP/RT group, mild rash occurred in two patients, which was caused by mild allergic reactions. A previous study showed that patients with locoregionally advanced nasopharyngeal carcinoma had good tolerance for h-R3/RT [27]. In our study, h-R3/RT did not aggravate the acute radiation reactions.

For sub-analyses, we analyzed the survivals of stage II patients in the h-R3/RT and CDDP/RT groups and found no significant difference in OS and PFS rates, likely suggesting that local control of the disease is mainly conveyed by radiation, without any correlation with the synchronous drugs [28]. Similar to the results above, stage II patients in the h-R3/RT group experienced less toxic effects. Moreover, among the patients aged more than 60 years, there was no significant difference in survival between the h-R3/RT and CDDP/RT groups, and less toxic effects were found in the h-R3/RT group. Therefore, we can reasonably believe that h-R3/RT is a better choice for stage II patients and patients aged more than 60 years. Previous studies of head and neck carcinomas and nasopharyngeal carcinoma have indicated that concurrent chemoradiotherapy is more effective than radiotherapy alone. However, there were no patients who received radiotherapy alone in our study, and thus we cannot compare the differences between concurrent chemoradiotherapy and radiotherapy alone. It was previously reported that the 5-year OS rate of stage II nasopharyngeal carcinoma patients treated with concurrent chemoradiotherapy compared to radiotherapy alone was 94.5% vs. 85.8%, respectively [29]. Concurrent chemoradiotherapy is feasible and effective in elderly patients with locoregionally advanced NPC who are not troubled with any severe comorbidities [30, 31]. Conclusively, our results suggested h-R3/RT as a alternative regimen, which not only guaranteed efficacy but also reduced side effects. Owing to our retrospective study and its relatively small sample size, this treatment provides only a potential remedy for concurrent radiotherapy of NPC in some specific patients.

The main prognostic factors are age, gender, clinical stage (T, N category included); the survival rate has been shown to decrease with the increasing T category and N category patients [32]. According to our data, T category, N category, clinical stage, vomit and synchronous drug were suspected to affect patients’ survivals according to the univariate COX analysis. The multivariate COX analysis indicated age, N category, and vomiting as independent prognostic factors. In contrast, gender was not significant in the analyses, which might have been a consequence of the small sample size and an underpowered analysis. Then, we analyzed the data of 302 patients using the methods mentioned above, and found that gender is a prognostic factor of nasopharyngeal carcinoma (Additional file 1: Table S8 and Additional file 1: Table S9). We observed that the synchronization of different drugs induced different outcomes: better survival was observed for the CDDP/RT group, which may be associated with the cytotoxicity of cisplatin. Moreover, h-R3/RT in nasopharyngeal carcinoma was may not improve long-term survival [3, 24]. Local recurrence and distant metastasis result in failed nasopharyngeal carcinoma treatment [33]. Moreover, we also analysised At the median follow-up of 50 months in our study, two patient had locoregional failure, two patient showed locoregional failure and distant metastases, and ten patients developed distant metastases. The sites of recurrence were mainly bone, lung and liver, consistent with previous reports in the literature [34].

It is worth mentioning that role of neoadjuvant chemotherapy has been questioned and evaluated in many ways [35, 36]. Most of the proponent opinions on neoadjuvant chemotherapy were based on the patients with stages III or IV. The cases in this study included patients with stages II, III and IV from September 2008 to November 2013 at West China Hospital Sichuan University. During the period of follow-up time, neoadjuvant chemotherapy (category 3) was recommended for patients with stage II by NCCN guidelines of Head and Neck Cancers (Additional file 1: Table S6). Furthermore, we analyzed the patients with stages III-IV. CDDP/RT patients achieved better survival (*p* = 0.009), while h-R3/RT patients experienced less leukopenia and milder nausea and vomiting, the *p* values were respectively 0.022 and 0.000 (Additional file 2: Figure S3, Additional file 1: Table S3). The results are similar to that obtained from 104 patients with stages II, III or IV. Although the role of neoadjuvant chemotherapy has been controversial according to recent literatures, it still remains to be explored. Our results, to some extent, provided direction for the therapeutic strategy of nasopharyngeal carcinoma. Meanwhile, we are expecting more powerful results about neoadjuvant chemotherapy, which may have a greater research value and application prospect in the future.

The results presented here must be interpreted cautiously because of the retrospective nature of this study and the small sample size. First, numerous factors are considered when determining the type of synchronous drugs for patients, most of which could not be captured in a retrospective medical record review, such as the economic condition of the patients. Second, as in many retrospective analyses, missing data were common. We may not have accounted for some confounding factors. Finally, some patients might have chosen other treatments, such as cell therapy, Chinese medicine treatment or other non-chemotherapy-based clinical trials, which could have limited the generalizability of these results. Owing to our retrospective study and its relatively small sample size, some prospective, randomized, well-designed, and large sample clinical studies are warranted to confirm these indications.

## Conclusions

Our findings suggest that concurrent administration of h-R3/RT might be a selectable strategy against nasopharyngeal carcinoma, although CDDP/RT remained the preferred choice for most patients with nasopharyngeal carcinoma. The regimen of h-R3/RT may be considered less toxic for patients with nasopharyngeal carcinoma, especially for some patients who do not well tolerate cisplatin, patients with stage II NPC and older patients. More effective and tolerable treatment regimens should be explored to improve survival rates and reduce the side-effects of patients with nasopharyngeal carcinoma. We are looking forward to prospective, well-designed, and large sample clinical studies.

## Additional files

Additional file 1: Table S1. Prognostic factors for overall survival (Univariate) (*N* = 104). **Table S2.** Prognostic factors for overall survival (multivariable) (*N* = 104). **Table S3.** Toxicities in stage III and IV patients with h-R3/RT and CDDP/RT (*N* = 78). **Table S4.** Assignment expressions for factors in the table of patients’ characteristics. **Table S5.** Patients’ compliance (104 cases). **Table S6.** Neoadjuvant chemotherapy was recommended by NCCN guidelines of Head and Neck Cancer. **Table S7.** General information for all 302 patients of CDDP/RT and h-R3/RT group. **Table S8.** Prognostic factors for Overall Survival of all 302 patients (Univariate). **Table S9.** Prognostic factors for Overall Survival of all 302 patients (Multivariable). (ZIP 437 kb)
Additional file 2: Figure S1. Overall survival of stage II patients who received h-R3/RT or CDDP/RT. **Figure S2.** Overall survival of patients aged more than 60 years old who received h-R3/RT or CDDP/RT. **Figure S3.** Overall survival in stage III and IV patients with h-R3/RT and CDDP/RT. (ZIP 12 kb)

## Abbreviations

ALT: Alanine transaminase
AST: Aspartate aminotransferase
CCRT: Concurrent chemoradiotherapy
CDDP: Cisplatin
CDDP/RT: Cisplatin and radiotherapy
CI: Confidence interval
CTCAE: Chemotherapy common adverse reactions guide
ECOG: Eastern Cooperative Oncology Group
EGFR: Epidermal growth factor receptor
GGT: Gamma glutamyl transpeptidase
HB: Hemoglobin
HR: Hazard ratio
h-R3/RT: Nimotuzumab and radiotherapy
IMRT: Intensity-modulated radiation therapy
NCCN: National comprehensive cancer network
NPC: Nasopharyngeal carcinoma
OS: Overall survival
PFS: Progression-free survival
PLT: Platelet
RT: Radiotherapy
SE: Standard error
WBC: White blood count
