# Supplementary material for: A retrospective paired study: efficacy and toxicity of nimotuzumab versus cisplatin concurrent with radiotherapy in nasopharyngeal carcinoma
Source: BMC Cancer. 2016 Dec 13;16:946. doi: 10.1186/s12885-016-2974-x (PMC5154088; doi:10.1186/s12885-016-2974-x)
Supplement: Additional file 1: Table S1. — Prognostic factors for overall survival (Univariate) (N = 104). Table S2. Prognostic factors for overall survival (multivariable) (N = 104). Table S3. Toxicities in stage III and IV patients with h-R3/RT and CDDP/RT (N = 78). Table S4. Assignment expressions for factors in the table of patients’ characteristics. Table S5. Patients’ compliance (104 cases). Table S6. Neoadjuvant chemotherapy was recommended by NCCN guidelines of Head and Neck Cancer. Table S7. General information for all 302 patients of CDDP/RT and h-R3/RT group. Table S8. Prognostic factors for Overall Survival of all 302 patients (Univariate). Table S9. Prognostic factors for Overall Survival of all 302 patients (Multivariable). (ZIP 437 kb) [file 12885_2016_2974_MOESM1_ESM.zip › Table S8R6.docx]

Table S8. Prognostic factors for Overall Survival of all 302 patients (Univariate)

| Factors | B | SE | P Value | HR(95.0%CI) |
| --- | --- | --- | --- | --- |
| Age | 0.583 | 0.253 | 0.021* | 1.791(1.092-2.938) |
| Sex | -0.099 | 0.258 | 0.701 | 0.906(0.547-1.501) |
| T category | 0.294 | 0.118 | 0.013* | 1.342 (1.065-1.692) |
| N category | 0.212 | 0.146 | 0.148 | 1.236(0.928-1.646) |
| M category | 0.823 | 0.428 | 0.055 | 2.277(0.983-5.270) |
| Clinical stage | 0.573 | 0.188 | 0.002* | 1.774(1.228-2.563) |
| WBC | -0.148 | 0.137 | 0.280 | 0.865(0.659-1.128) |
| PLT | -0.214 | 0.161 | 0.185 | 0.807(0.588-1.108) |
| HB | 0.279 | 0.181 | 0.123 | 1.321(0.927-1.882) |
| ALT | 0.308 | 0.242 | 0.203 | 1.361(0.847-2.187) |
| AST | 0.105 | 0.343 | 0.760 | 1.110(0.567-2.175) |
| GGT | -0.216 | 0.305 | 0.480 | 0.806(0.443-1.466) |
| Dermatitis | 0.134 | 0.201 | 0.506 | 1.143(0.771-1.694) |
| Rash | 0.144 | 0.125 | 0.251 | 1.155 (0.903-1.477) |
| Mucositis | 0.013 | 0.108 | 0.904 | 1.013(0.819-1.253) |
| Taste change | 0.125 | 0.343 | 0.715 | 1. 134(0.578-2.222) |
| Vomit | 0.589 | 0.104 | 0.000* | 0.555(0.453-0.680) |
| Weight loss | -0.058 | 0.120 | 0.630 | 0.944(0.746-1.194) |
| Drug | 0.153 | 0.292 | 0.599 | 1.166(0.658-2.064) |

*Abbreviations*: HR, hazard ratio; CI, confidence interval; SE, standard error; B, partial regression coefficient.
